# Supplementary material for: Modeling glioblastoma heterogeneity as a dynamic network of cell states
Source: Mol Syst Biol. 2021 Sep 16;17(9):e10105. doi: 10.15252/msb.202010105 (PMC8444284; doi:10.15252/msb.202010105)
Supplement: Supplementary file 6 — Source Data for Figure 5 [file MSB-17-e10105-s004.zip › Figure5A_sourcedata/GSEA_3071/hallmarks_stateVI.GseaPreranked.1623742557031/gsea_report_for_na_pos_1623742557031.html]

Report for na\_pos 1623742557031 [GSEA]

| GS  follow link to MSigDB | GS DETAILS | SIZE | ES | NES | NOM p-val | FDR q-val | FWER p-val | RANK AT MAX | LEADING EDGE || 1 | HALLMARK\_TNFA\_SIGNALING\_VIA\_NFKB | Details ... | 87 | 0.35 | 2.49 | 0.000 | 0.000 | 0.000 | 578 | tags=45%, list=16%, signal=52% |
| 2 | HALLMARK\_MYC\_TARGETS\_V2 | Details ... | 40 | 0.43 | 2.46 | 0.000 | 0.000 | 0.000 | 1723 | tags=90%, list=47%, signal=169% |
| 3 | HALLMARK\_HYPOXIA | Details ... | 79 | 0.34 | 2.37 | 0.000 | 0.000 | 0.000 | 473 | tags=37%, list=13%, signal=41% |
| 4 | HALLMARK\_UNFOLDED\_PROTEIN\_RESPONSE | Details ... | 37 | 0.41 | 2.34 | 0.000 | 0.000 | 0.000 | 1531 | tags=81%, list=42%, signal=139% |
| 5 | HALLMARK\_MYC\_TARGETS\_V1 | Details ... | 98 | 0.32 | 2.31 | 0.000 | 0.000 | 0.000 | 1135 | tags=57%, list=31%, signal=81% |
| 6 | HALLMARK\_UV\_RESPONSE\_DN | Details ... | 63 | 0.35 | 2.21 | 0.000 | 0.001 | 0.002 | 192 | tags=29%, list=5%, signal=30% |
| 7 | HALLMARK\_INFLAMMATORY\_RESPONSE | Details ... | 46 | 0.37 | 2.18 | 0.000 | 0.002 | 0.005 | 267 | tags=33%, list=7%, signal=35% |
| 8 | HALLMARK\_APICAL\_JUNCTION | Details ... | 61 | 0.30 | 1.93 | 0.007 | 0.013 | 0.037 | 673 | tags=39%, list=18%, signal=47% |
| 9 | HALLMARK\_EPITHELIAL\_MESENCHYMAL\_TRANSITION | Details ... | 99 | 0.27 | 1.90 | 0.000 | 0.014 | 0.047 | 335 | tags=29%, list=9%, signal=31% |
| 10 | HALLMARK\_MTORC1\_SIGNALING | Details ... | 77 | 0.24 | 1.68 | 0.010 | 0.042 | 0.143 | 989 | tags=51%, list=27%, signal=68% |
| 11 | HALLMARK\_ALLOGRAFT\_REJECTION | Details ... | 35 | 0.31 | 1.66 | 0.017 | 0.042 | 0.161 | 889 | tags=46%, list=24%, signal=60% |
| 12 | HALLMARK\_IL2\_STAT5\_SIGNALING | Details ... | 64 | 0.25 | 1.63 | 0.006 | 0.045 | 0.186 | 1182 | tags=56%, list=32%, signal=82% |
| 13 | HALLMARK\_REACTIVE\_OXYGEN\_SPECIES\_PATHWAY | Details ... | 16 | 0.40 | 1.63 | 0.028 | 0.042 | 0.188 | 1170 | tags=75%, list=32%, signal=110% |
| 14 | HALLMARK\_TGF\_BETA\_SIGNALING | Details ... | 29 | 0.29 | 1.48 | 0.078 | 0.093 | 0.390 | 98 | tags=17%, list=3%, signal=18% |
| 15 | HALLMARK\_XENOBIOTIC\_METABOLISM | Details ... | 38 | 0.25 | 1.41 | 0.072 | 0.127 | 0.515 | 422 | tags=26%, list=12%, signal=29% |
| 16 | HALLMARK\_INTERFERON\_GAMMA\_RESPONSE | Details ... | 44 | 0.23 | 1.37 | 0.116 | 0.146 | 0.589 | 962 | tags=43%, list=26%, signal=58% |
| 17 | HALLMARK\_ESTROGEN\_RESPONSE\_EARLY | Details ... | 58 | 0.21 | 1.36 | 0.082 | 0.139 | 0.591 | 600 | tags=31%, list=16%, signal=37% |
| 18 | HALLMARK\_ANDROGEN\_RESPONSE | Details ... | 33 | 0.25 | 1.34 | 0.148 | 0.146 | 0.639 | 256 | tags=21%, list=7%, signal=23% |
| 19 | HALLMARK\_OXIDATIVE\_PHOSPHORYLATION | Details ... | 61 | 0.21 | 1.34 | 0.054 | 0.138 | 0.640 | 1498 | tags=62%, list=41%, signal=104% |
| 20 | HALLMARK\_WNT\_BETA\_CATENIN\_SIGNALING | Details ... | 16 | 0.31 | 1.26 | 0.194 | 0.196 | 0.785 | 143 | tags=19%, list=4%, signal=19% |
| 21 | HALLMARK\_FATTY\_ACID\_METABOLISM |  | 33 | 0.23 | 1.25 | 0.196 | 0.193 | 0.798 | 596 | tags=33%, list=16%, signal=40% |
| 22 | HALLMARK\_ADIPOGENESIS |  | 48 | 0.19 | 1.14 | 0.246 | 0.297 | 0.927 | 1082 | tags=46%, list=30%, signal=64% |
| 23 | HALLMARK\_GLYCOLYSIS |  | 65 | 0.17 | 1.12 | 0.271 | 0.317 | 0.945 | 473 | tags=25%, list=13%, signal=28% |
| 24 | HALLMARK\_INTERFERON\_ALPHA\_RESPONSE |  | 18 | 0.18 | 0.77 | 0.782 | 0.791 | 1.000 | 2989 | tags=100%, list=82%, signal=557% |
Table: Gene sets enriched in phenotype **na **[plain text format]****
